# Supplementary material for: Simultaneous presence of Mycoplasma salivarium and Tannerella forsythia in the implant sulcus after lateral augmentation with autogenous root grafts is associated with increased sulcus probing depth
Source: PLoS One. 2022 Jul 8;17(7):e0270962. doi: 10.1371/journal.pone.0270962 (PMC9269361; doi:10.1371/journal.pone.0270962)
Supplement: S1 Table — (DOCX) [file pone.0270962.s001.docx]

**S1 Table:**

| **Bacteria** | Gene | Primers /Probe | Sequences (5´-3´) |
| --- | --- | --- | --- |
| *Eubacteria* | 16S rDNA | Euba-F | TGGAGCATGTGGTTTAATTCGA |
|  |  | Euba-R | TGCGGGACTTAACCCAACA |
|  |  | Euba-S | TexRed-CACGAGCTGACGACARCCATGCA-BHQ2 |
| *Mycoplasma salivarium* | *rpo*B | msali-F | CCGTCAAATGATTTCGATTGC |
|  |  | msali-R | GAACTGCTTGACGTTGCATGT T |
|  |  | msali-S | HEX-ATATGCTAACCGTGCGCTTATGGGTG-BHQ1 |
| *Veillonella parvula* | 16S rDNA | vpar-F | TGCTAATACCGCATACGATCTAACC |
|  |  | vpar-R | GCTTATAAATAGAGGCCACCTTTCA |
|  |  | vpar-S | HEX-CTATCCTCGATGCCGA-BHQ1 |
| *Staphylococcus aureus* | *nuc* | saur-F | CAAAGCATCCTAAAAAAGGTGTAGAGA |
|  |  | saur-R | TTCAATTTTCTTTGCATTTTCTACCA |
|  |  | saur-S | FAM-TTTTCGTAAATGCACTTGCTTCAGGACCA-BHQ1 |
| *Porphyromonas* | Arg- | pgin-F | CCTACGTGTACGGACAGAGCTATA |
| *gingivalis* | gingiplain | pgin-R | AGGATCGCTCAGCGTAGCATT |
|  |  | pgin-S | TexRed-TCGCCCGGGAAGAACTTGTCTTCA-BHQ2 |
| *Parvimonas micra* | 16S rDNA | pmic-F | TCGAACGTGATTTTTGTGGAAA |
|  |  | pmic-R | GGTAGGTTGCTCACGTGTTACTCA |
|  |  | pmic-S | FAM-CCCGTTCGCCACTT-BHQ1 |
| *Tannerella forsythia* | *bsp*A | tfor-F | TCCCAAAGACGCGGATATCA |
|  |  | tfor-R | ACGGTCGCGATGTCATTGT |
|  |  | tfor-S | FAM-CCGCGACGTGAAATGGTATTCCTC-BHQ1 |
|  |  | tfor-S II | HEX-TCGCGACGTGAAATGGTATTCCTC-BHQ1 |

**Overview of bacterial species, corresponding genes, primers/probes, and DNA sequences as published [26]**
