# Supplementary material for: Simultaneous presence of Mycoplasma salivarium and Tannerella forsythia in the implant sulcus after lateral augmentation with autogenous root grafts is associated with increased sulcus probing depth
Source: PLoS One. 2022 Jul 8;17(7):e0270962. doi: 10.1371/journal.pone.0270962 (PMC9269361; doi:10.1371/journal.pone.0270962)
Supplement: S2 Table — Group 1: cortical autogenous bone blocks (CABB); Group 2: healthy autogenous tooth roots (HATR); Group 3: roots from non-preservable teeth (NPTR); t1: beginning of the prosthetic restauration, t2: six months after completing of the prosthetic restauration. (DOCX) [file pone.0270962.s002.docx]

|  | **t1** | | | **t2** | | |
| --- | --- | --- | --- | --- | --- | --- |
|  | **Group 1/**  **CABB**  **(n=11)** | **Group 2/**  **HATR**  **(n=14)** | **Group 3/**  **NPTR**  **(n=13)** | **Group 1/**  **CABB**  **(n=11)** | **Group 2/**  **HATR**  **(n=14)** | **Group 3/**  **NPTR**  **(n=13)** |
| **Tooth** | 4 | 11 | 5 | 6 | 6 | 5 |
| **Implant** | 1 | 7 | 4 | 7 | 12 | 5 |

**S3 Table: Number of *Mycoplasma salivarium* positive submucosal biofilm and peri-implant sulcus fluid samples by underlying augmentation material by intervention group.**

Group 1: cortical autogenous bone blocks (CABB); Group 2: healthy autogenous tooth roots (HATR); Group 3: roots from non-preservable teeth (NPTR); t1: beginning of the prosthetic restauration, t2: six months after completing of the prosthetic restauration.
