# Supplementary figures and images for: Simultaneous presence of Mycoplasma salivarium and Tannerella forsythia in the implant sulcus after lateral augmentation with autogenous root grafts is associated with increased sulcus probing depth
Source: PLoS One. 2022 Jul 8;17(7):e0270962. doi: 10.1371/journal.pone.0270962 (PMC9269361; doi:10.1371/journal.pone.0270962)

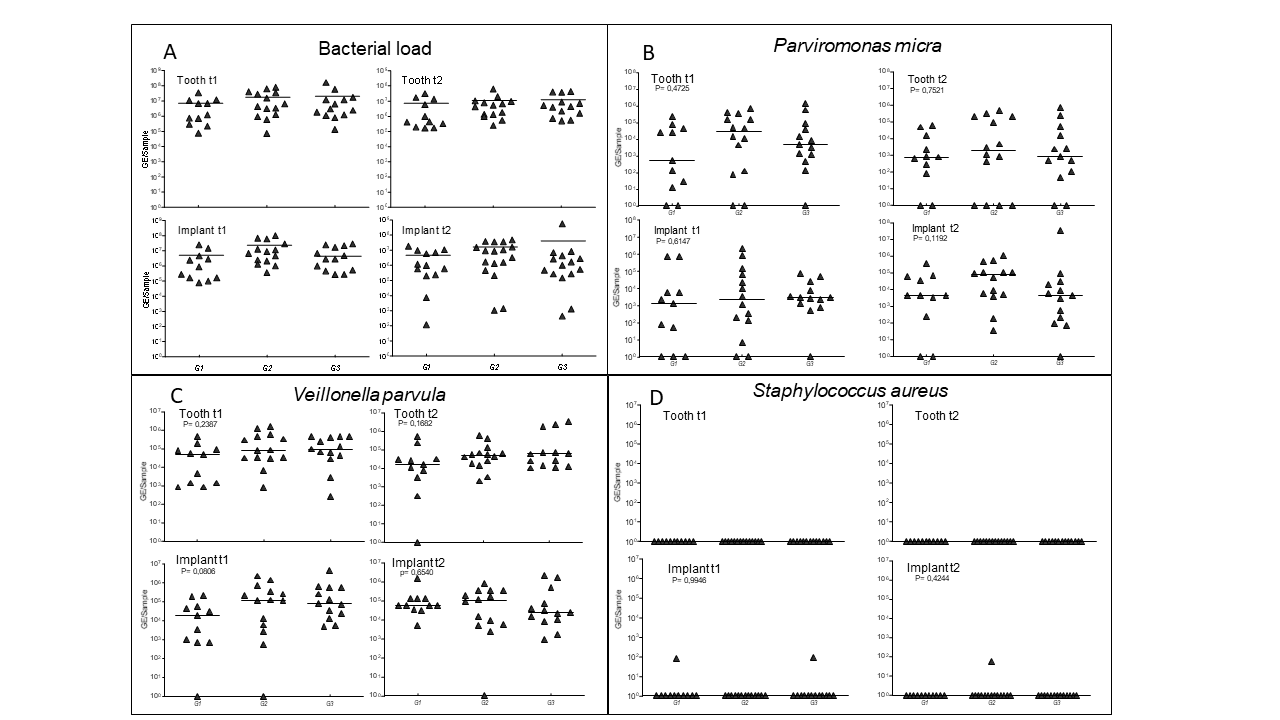

Supplement: S1 Fig — Scatter plots of the bacterial quantity of (A) the Bacterial load, (B) Parviromonas micra, (C) Veillonella parvula and (D) Staphylococcus aureus indicated as genome equivalents per sample (GE/sample) in the peri-implantary sulcus compared to the opposite tooth, over time in the different augmentation groups. G1: Group 1, cortical autogenous bone blocks; G2: Group 2, healthy autogenous tooth roots; G3: Group 3, roots from non-preservable tooth; t1: begin of the prosthetic restauration; t2: six months after completing of the prosthetic restauration The bars represent mean. (TIF) [file pone.0270962.s003.tif]
