# Supplementary material for: Simultaneous presence of Mycoplasma salivarium and Tannerella forsythia in the implant sulcus after lateral augmentation with autogenous root grafts is associated with increased sulcus probing depth
Source: PLoS One. 2022 Jul 8;17(7):e0270962. doi: 10.1371/journal.pone.0270962 (PMC9269361; doi:10.1371/journal.pone.0270962)
Supplement: Attachment — Submitted filename: Msali_pbp_reply.docx [file pone.0270962.s005.docx]

Dear Professor Eickholz,

We thank the Editorial Office and the Reviewers for their kind assessment of our manuscript and the constructive and helpful feedback. We feel that addressing the issues raised helped us to clarify certain points in the manuscript and to improve the strength of the statistical analysis. Please find our replies (in *italics*) to the comments and suggestions below. Line numbers for corresponding changes refer to the numbering in the tracked-changes version of the revised manuscript.

Editorial office:

a) If there are ethical or legal restrictions on sharing a de-identified data set, please explain them in detail (e.g., data contain potentially sensitive information, data are owned by a third-party organization, etc.) and who has imposed them (e.g., an ethics committee). Please also provide contact information for a data access committee, ethics committee, or other institutional body to which data requests may be sent.

*> Under the European Union’s General Data Protection Regulation, handling and publication of any non-anonymised protected data is subject to the individual’s consent. Consent for public sharing of individual level data has not been obtained from study participants. However, after careful consideration of the data used for analyses for the current report, we conclude that complete anonymisation was indeed possible by omitting the pseudonymised study identifiers and randomly assigning new patient level identifiers. We now include this anonymised dataset as a supplementary file.*

b) If there are no restrictions, please upload the minimal anonymized data set necessary to replicate your study findings as either Supporting Information files or to a stable, public repository and provide us with the relevant URLs, DOIs, or accession numbers. For a list of acceptable repositories, please see http://journals.plos.org/plosone/s/data-availability#loc-recommended-repositories.

We will update your Data Availability statement on your behalf to reflect the information you provide.

*> As explained above, we now provide an anonymised dataset.*

3. We note that you have included the phrase “data not shown” in your manuscript. Unfortunately, this does not meet our data sharing requirements. PLOS does not permit references to inaccessible data. We require that authors provide all relevant data within the paper, Supporting Information files, or in an acceptable, public repository. Please add a citation to support this phrase or upload the data that corresponds with these findings to a stable repository (such as Figshare or Dryad) and provide and URLs, DOIs, or accession numbers that may be used to access these data. Or, if the data are not a core part of the research being presented in your study, we ask that you remove the phrase that refers to these data.

*> We thank the Office for highlighting this. Data supporting these statements has now been included in the supplements and the respective phrases in the manuscript have been adapted.*

*Corresponding changes:*

*Line 175*

*Line 199*

4. Please include captions for your Supporting Information files at the end of your manuscript, and update any in-text citations to match accordingly. Please see our Supporting Information guidelines for more information: http://journals.plos.org/plosone/s/supporting-information.

*> Captions for the supplementary files have now been included at the end of the manuscript.*

Reviewer #1: The purpose of this research study was to characterize the pathogenic role of Mycoplasma salivarium and bacterial co-detection patterns on different implant augmentation types. The conclusions are unclear.

Major revision:

A more sophisticated statistical analysis that accounts for the correlation among repeated measures within a patient is needed. Perhaps a mixed linear regression model with factors for time, treatment group, and side would be more appropriate.

*> We thank the Reviewer for this important suggestion. Delineating interfering random effects on the levels of the variables suggested by the Reviewer from the fixed effects estimated by the model we had included is indeed an important addition. This refers to the second set of analyses in the report, where we pool samples from different groups, sides and timepoints for overall association analyses. Based on the data presented for the first analyses, which are separated by group, side and timepoint, we proposed that random effects would be negligible. We do, however, agree that a more formal assessment of this proposal is warranted. In response to the Reviewer’s suggestion, we now include estimates from a multilevel mixed-effects model with nested random effects for (from highest to lowest level): sampling (tooth or implant), treatment group and timepoint of sampling. The resulting estimates for the fixed effects were indeed very close to the ones from the previous model, indicating that random effects on the variables mentioned did not interfere with the fixed-effects estimations.*

*Corresponding changes:*

*Lines 159 – 164*

*Lines 244 – 247*

Minor revisions:

1- Line 149: “Across” treatment groups.

*> This has now been corrected.*

2- State and justify the study’s target sample size with a pre-study statistical power calculation. The power calculation should include: sample size, alpha level (indicating one or two-sided), minimal detectable difference and statistical testing method.

*> The Reviewer raises an important point. Although the setting of the study as a sub-study within an interventional trial was implied in our manuscript, we did not explain this explicitly and we agree that this might be confusing for the reader. The sample-size calculation was strictly performed for the interventional trial and the underlying assumptions have been detailed in the published report for this trial. The sub-study we report on here was designed as exploratory and made secondary use of the sample recruited for the trial, without a pre-specified statistical analysis plan. Obviously, this design carries risks. However, due to the scarcity of previous data on the subject and the need to adapt to the main trial, this was the only feasible design. Incorporating exploratory sub-studies on unexplored questions into interventional trials is common and good practice. These sub-studies have the aim to clarify hypotheses and lead to subsequent, more focused studies. We believe that the study we report on meets this goal, but we do agree that the exploratory nature of the study needed better explanation.*

*Corresponding changes:*

*Lines 149 - 151*

3- The p-value associated with a correlation is a test of the null hypothesis: correlation equal to zero; however, the absolute magnitude of the coefficient indicates the strength of the linear relationship between two variables. In general, the strength or correlation coefficient is the more important statistic to focus on.

Below is a table for interpreting correlation coefficients:

Coefficient (absolute value) Interpretation

0.90 - 1.0 Very Strong

0.70 - 0.89 Strong

0.40 - 0.69 Moderate

0.10 - 0.39 Weak

less than 0.10 Negligible correlation

*> We thank the Reviewer for providing this frame for reference and adapted the wording in the manuscript accordingly.*

*Corresponding changes: Throughout the paragraphs from line 223 to line 261.*

Reviewer #2: I have some problems with the major purpose of the study. As there is obviously nothing known about the microbial colonization regarding augmentation, I would not set the primary focus on a bacterial species which was only very rarely described.

*> We agree with the reviewer that the focused interest in exploring interactions of Mycoplasma salivarium may seem uncommon. The senior investigator of the sub-study, Prof. Henrich, dedicated more than 20 years of her academic career to the investigation of roles of Mycoplasma spp. As potential pathogens. Europe’s largest scientific society for clinical microbiology, the European Society for Clinical Microbiology and Infectious Diseases, acknowledges the importance of this work by having formed a dedicated Mycoplasma Research Group. We provide detailed information on the (admittedly few) previous reports on M. salivarium’s role as a possible oral pathogen in the introduction and discussion sections of the manuscript. Although other potential pathogens may feature more prominently in the scientific literature, we believe that exploring M. salivarium’s role is important.*

Please use the actual term “biofilm” (instead of plaque).

> The wording in the manuscript has been changed accordingly.

*Corresponding changes: Throughout the manuscript.*

Although, analysis of microbiota was obviously not the primary aim of the study, please mention the primary outcome and the respective power analysis.

*> In line with our response to Reviewer 1, a power or sample size calculation for the sub-study based on pre-analysis considerations is not possible due to the exploratory design.*

Why did the authors focus on the selected microorganisms? I miss Treponema denticola at least. Please provide the detection level of each PCR.

*> Individual results for all microorganisms are now provided as part of the anonymised minimal dataset in the supplementary material.*

*The panel of microorganisms analysed for this study was the result of a previous study referenced in our report (number 26 in our reference list: Schwarz F, Becker K, Rahn S, Hegewald A, Pfeffer K, Henrich B. Real-time PCR analysis of fungal organisms and bacterial species at peri-implantitis sites. International journal of implant dentistry. 2015;1(1):9). We agree with the Reviewer that the panel is not comprehensive. Selection of microorganisms for the panel was based on the aim to include a set of microorganisms known to represent a spectrum of strengths of association with periodontal disease. However, as little is known about pathogens in peri-implant inflammation, even a complete set of microorganisms with strongly established roles as pathogens or indicators of periodontal inflammation would not necessarily be comprehensive. Any panel study can be correctly criticised in this way, and given the wider accessibility of metagenomic sequencing techniques, we believe that future studies, including our own subsequent work, need to employ bias-free or bias-reduced methods.*

The data on positive results of all analyzed microorganisms should be presented. In my suggestion, these data (although not being statistically significant different between the groups and over time) might be of interest.

*> We thank the Reviewer for this suggestion, data for all analysed microorganisms are now included in supplementary figure 2.*

The last sentence in the conclusion of the abstract should be weaken.

*> We agree with the Reviewer that a weaker wording more accurately describes the findings of the study and changed the sentence accordingly.*

Did the authors also collect peri-implant sulcular fluid for analysis of certain biomarkers in addition? If yes, inclusion of these data would increase the merit of that manuscript.

*> Although we agree that this is an excellent idea, we did not measure biomarkers in sulcular fluid and were not able to retain sufficient fluid volume to add these analyses now.*
