# Supplementary material for: Simultaneous presence of Mycoplasma salivarium and Tannerella forsythia in the implant sulcus after lateral augmentation with autogenous root grafts is associated with increased sulcus probing depth
Source: PLoS One. 2022 Jul 8;17(7):e0270962. doi: 10.1371/journal.pone.0270962 (PMC9269361; doi:10.1371/journal.pone.0270962)
Supplement: Attachment — Submitted filename: Msali_pbp_r2.docx [file pone.0270962.s006.docx]

**Point-by-point reply**

We thank the Reviewers for their insightful comments and suggestions, and their continuing support in improving the clarity of the manuscript.

Please find our point-by-point reply to the comments below. Line numbers refer to numbering in the tracked changes version of the revised manuscript.

Reviewer #1: Minor Revision:

Indicate the underlying covariance structure used in the mixed effects model and the criteria for selecting it.

- Because possible random effects are nested (i.e. on different levels) but also clearly independent (in that they cannot be associated with one another because the study design defines that both tooth and implant side are sampled at two timepoints in each treatment group, i.e. across these variables data is evenly distributed), selection of different covariance structures did not impact the fixed or random effect estimates. For additional clarity, an independent covariance structure has been specified in the manuscript now (l 168). Yet, using unstructured covariance creates the same results in the model.

Reviewer #2: The authors responded to my raised concerns. Answers to minor comments I accept. But still I do not understand the purpose of the study. A bacterium probably of importance in development of oral diseases was investigated related to three augmentation procedures. What is the rationale behind?

Studying the presence and quantity of Mycoplasma salivarium related to disease categories (healthy, mucositis, peri-implantitis, gingivitis, periodontitis) might be of interest. And still, including 36 patients in a clinical research project, would need a primary outcome.

- The primary outcome of the main trial that participants were included into is stated in reference number 17 of the current manuscript (“clinical width of the alveolar ridge”, operationalised as: “The primary endpoint was defined as the clinical width (CW) of the alveolar ridge being sufficient for the placement of an adequately dimensioned dental implant without the need for a secondary grafting at 26 weeks after surgery in either group.”). The nested study that we report on in this manuscript did not independently include patients. Rather, the patient population included in the main trial contributed samples used for this exploratory study. We believe that it is important to explain the intervention used in the main trial in some detail for this manuscript, as potential effects of this intervention are studied here. However, we decided not to elaborate on other aspects of the design of the main trial because they are of little consequence to this study and a reference for the main trial’s report is provided so that interested readers may find more detail there.

In our opinion, investigating abundance of *Mycoplasma salivarium* in different augmentation procedures makes best use of the background of how the respective samples for this study were obtained. We believe this approach links a broader question of potential roles of *M. salivarium* in peri-implant disease to a more specific clinical application by demonstrating that abundance of *M. salivarium* may not simply be something to be observed, but may be dependent on patient management, in this case the use of different augmentation methods. We aimed to interpret the findings with appropriate caution, given that treatment allocation was not randomised, yet there is an association between augmentation method and detection of *M. salivarium*, which we argue warrants further study.

Reviewer #3: This is the long term follow up of a RCT focusing on the efficacy of two different autologous tooth preparations as test group and bone blocks as control (Ref #17). This should be more clearly pointed out in the methods. The present study is no new study, but an important follow up.

- We thank the Reviewer for this suggestion. We added the requested clarification at appropriate points in the introduction and methods sections (ll 72-73, 81 and 94-95). Further, we now stringently refer to the interventional (main) study as “trial” which should also help to delineate mentioning of the trial and the sub-study reported on here.

As this is a sub group analysis with a descriptional pilot character, the importance of a power calculation from my point of view should not be overestimated.

The title is difficult to understand. I would suggest: “Simultaneous presence of Mycoplasma salivarium and Tannerella forsythia in the sulcus implants after lateral augmentation with autogenous root grafts is associated with increased sulcus probing depth.

- We adapted the title as suggested with a minor modification (“implant sulcus” instead of “sulcus implants”).

Page 2, line 27 & 32: „gingiva“ around implants the term “mucosa” would be more appropriate in the field of implantology.

- We changed the wording in the abstract accordingly.

Materials and Methods are clear and complete.

the results are presented in an appropriate manner.

The discussion addresses all important aspects and covers also the weakness of the study.

The conclusions are supported by the data.
